# Leveraging Multiple Distinct EEG Training Sessions for Improvement of Spectral-Based Biometric Verification Results

**DOI:** 10.3390/s23042057

**Published:** 2023-02-11

**Authors:** Renata Plucińska, Konrad Jędrzejewski, Urszula Malinowska, Jacek Rogala

**Affiliations:** 1 Institute of Electronic Systems, Faculty of Electronics and Information Technology, Warsaw University of Technology, 00-665 Warsaw, Poland; 2 Institute of Experimental Physics, Faculty of Physics, University of Warsaw, 02-093 Warsaw, Poland

**Keywords:** biometry, EEG, electroencephalography, neural network, PSD, verification

## Abstract

Most studies on EEG-based biometry recognition report results based on signal databases, with a limited number of recorded EEG sessions using the same single EEG recording for both training and testing a proposed model. However, the EEG signal is highly vulnerable to interferences, electrode placement, and temporary conditions, which can lead to overestimated assessments of the considered methods. Our study examined how different numbers of distinct recording sessions used as training sessions would affect EEG-based verification. We analyzed the original data from 29 participants with 20 distinct recorded sessions each, as well as 23 additional impostors with only one session each. We applied raw coefficients of power spectral density estimate, and the coefficients of power spectral density estimate converted to the decibel scale, as the input to a shallow neural network. Our study showed that the variance introduced by multiple recording sessions affects sensitivity. We also showed that increasing the number of sessions above eight did not improve the results under our conditions. For 15 training sessions, the achieved accuracy was 96.7 ± 4.2%, and for eight training sessions and 12 test sessions, it was 94.9 ± 4.6%. For 15 training sessions, the rate of successful impostor attacks over all attack attempts was 3.1 ± 2.2%, but this number was not significantly different from using six recording sessions for training. Our findings indicate the need to include data from multiple recording sessions in EEG-based recognition for training, and that increasing the number of test sessions did not significantly affect the obtained results. Although the presented results are for the resting-state, they may serve as a baseline for other paradigms.

## 1. Introduction

Hans Berger was the first to record and publish a paper on electroencephalography (EEG) signals [1]. Since then, the interest in using bioelectrical signals from the head surface has grown in popularity. Over the years, technical achievements and improvements in the quality of EEG-collecting devices and signal analysis methods have enabled the use of EEG in many fields.

One of the advantages of the EEG signals is that they can be non-invasively acquired by placing electrodes on the surface of the subject’s head, which allows the determination of the distribution of bioelectrical activity in the brain. The resulting data are expressed as a function of voltage over time. In addition, this method is relatively inexpensive and has the highest time resolution compared to other techniques for examining brain function. It is widely used in various medical areas, as well as in EEG–neurofeedback training [2], emotion recognition [3], and neuroscience research. It allows for the acquisition of information on cognitive functions [4], sleep patterns [5], neurological disorders like epilepsy [6,7], or the depth of anesthesia [8]. Machine learning (ML) and neural network (NN) methods are often used to analyze EEG signals. They have been successfully applied to identify patterns of attention deficit hyperactivity disorder (ADHD) [9] and mental disorders such as schizophrenia [10] based on EEG analysis. There is also the potential to apply these methods for EEG-based people recognition.

Biometric recognition is the “automated recognition of individuals based on their biological and behavioural characteristics” [11] which can be required by security or access control systems. The most popular biometric characteristics are based on fingerprints, faces, irises, retinas, blood vessels, handwriting, gait, and voice. Research on using EEG signals as biometric characteristics has been ongoing for years [12,13,14,15,16,17,18]. The main advantage of EEG as a biometric is its hidden nature which makes it less vulnerable to attack by impostors or intruders compared to more popular modalities [19,20]. Since the brain of a deceased person cannot generate EEG signals, these signals can only be acquired from a living being, making the data more difficult to counterfeit. Moreover, these data cannot be captured at a distance [21]. EEG can be used for verification—which is the confirmation of a claimed identity, and identification—which involves searching for a person in a database of authorized individuals and returning a match [11]. The use of EEG in biometrics is based on the fact that brainwave patterns have large inter-personal differences between people and small intra-personal differences [22].

The EEG-based recognition systems can be classified according to the acquisition protocol into those based on external stimulation protocols, internal stimulation protocols, and resting-state protocols with eyes open (REO) or eyes closed (REC) [23]. In EEG biometric systems based on external stimulation [15,17], the participant is presented with a stimulus (such as visual, auditory, or somatosensory) and is then recognized based on the unique evoked response. Protocols using internal stimulation [24] require the full cooperation of the participant who is asked to perform some actions mostly related to, for example, thinking, imagining something, or calculations. The resulting EEG potentials are then analyzed. While both external and internal stimulation paradigms have shown promising results, they require the complete attention of the participant and are not suitable for continuous authentication. In addition, both paradigms require further, more comprehensive research on the permanence of the solutions and whether the subject becomes bored after repeated exposure to multiple stimuli or performing many mental tasks over time.

One of the benefits of EEG-based verification using resting-state data is that it does not require engagement from the test subject, and it is impossible to become used to it over time. It primarily relies on signal spectral features and artificial neural networks [25]. However, signals recorded in the resting-state paradigm are more challenging to integrate the obtained information due to nonstationary changes induced by the spontaneous activity, which causes covariance shifts of the power features [26,27].

Due to the nature of the EEG, resting-state EEG-based [22,28,29,30] verification with eyes open may find its place in a different application than other biometrics, like fingerprints, for example. It can be used in systems that require continuous identity confirmation, in brain-computer interfaces, or in EEG neurofeedback therapies based on participant-specific protocols where the subject must be verified to have the particular protocol applied. By using EEG biometrics, the verification and application of suitable protocols might be seamlessly integrated. Due to its specificity, the EEG can be used in access control systems in places requiring a high level of security; e.g., in military or government systems. Moreover, resting-state EEG can be collected from the mentally ill or from those unable to perform simple tasks, making it easier to obtain than other EEG-based recognition paradigms. Another advantage of the EEG-based methods over other biometrics methods is its sensitivity to excessive stress, which may prevent entry attempts during a hostage situation. However, EEG collected with medical-grade helmets requires a long preparation process for data acquisition [18], and well-described and verified analytical methods, including the system assessment based on multiple distinct acquisition sessions performed on different days [31], are lacking.

The foundation for EEG-based biometrics was established in earlier studies [12,13,14]. In these experiments, using only one channel (O2-Cz), the authors obtained satisfactory results for four subjects with 45 sessions each and 75 people with one session each. In one study [12], the data from overlapping sub-bands in the alpha band (7–10 Hz, 8–11 Hz, 9–12 Hz) from REC signals were analyzed using the FFT (Fast Fourier Transform) and neural network classifier. The authors analyzed two test cases; one for verification and the second for identification. In the first one, they verified the identity of four individuals and obtained Correct Classification scores ranging from 80% to 100% depending on the analyzed sub-band and individual.

In 2010, researchers [16] used features extracted with wavelet packet decomposition and a neural network to achieve the Correct Recognition Rate (CRR) of 81% based on four channels of EEG and 71% for two channels of EEG. The studies were conducted on a group of 10 participants with five separate sessions conducted over two weeks. The results were presented for REO and REC signals.

An attempt was made to evaluate the impact of the number of distinct recording sessions on the recognition results [31]. The authors analyzed the permanence of a database of 45 healthy adults with five to six distinct recording sessions each, collected over three years. They analyzed the Event-Related Potential (ERP) EEG and used the Hidden Markov Models as classifiers. They pointed out that using only one recording session to perform EEG-based recognition may lead to overestimating the results.

Most studies seem to focus more on analyzing inter-individual differences than their permanence [21]. Some studies have attempted to address this issue by increasing the number of distinct recording sessions and analyzed data from five to six sessions [16,31,32]. However, many studies are developing complex algorithms based on data from one recording session [15,22,24,33,34,35,36,37]; or on data from more sessions but performed on the same day [38]; or with a limited number of people with multiple distinct sessions [12,13,14]. Therefore, there is a strong need for more comprehensive research on the impact of the number of recording distinct sessions on the permanence of EEG-based recognition [23]. The subject is still of interest, as evidenced by recently published articles on the use of EEG in biometrics [19,25,31,32,39,40].

Studies reported that the popular methods for extraction of the EEG features include Time-Series Models such as Autoregressive Models, Connectivity-Based Methods, and Frequency-Based Methods, mostly based on Power Spectral Density (PSD) or Wavelet Transform [23,40]. Moreover, others [23] described methods based on Cosine Transform and Mel-Frequency Cepstrum, and other methods based on supervised learning, such as Linear Discriminant Analysis (LDA), Common Spatial Pattern (CSP), and NN. They also described unsupervised learning methods such as Statistical Methods (Principal Component Analysis, Independent Component Analysis), Clustering-Based Methods, Restricted Boltzmann Machines, Autoencoders, and Generative Adversarial Networks. In these papers [23,40], the classification methods were reported, such as k-Nearest Neighbors, Neural Networks, Kernel Methods (e.g., Support Vector Machine), and Deep Learning.

Our studies aimed to evaluate how the number of distinct EEG recording sessions would affect the verification results. Taking into account the results of our previous studies [41] and studies performed by other authors [12,35,36], we decided to focus on the PSD coefficients. Because we tried to simulate actual verification conditions, we did not process any artifact rejection.

## 2. Methodology

### 2.1. Data Collection

In the following, we analyzed 580 recordings originating from 29 adult participants (14 females and 15 males, with a mean age of 28.17 years, ranging from 23 to 44 years). For each participant, 20 EEG recording sessions were conducted over an average of approximately 70 days (ranging from 43 to 129 days). Additionally, we simulated an external attack by impostors using recordings from an additional 23 subjects who were not included in the previous analyses. In total, 603 examinations were used in the study.

The 19 electrodes were placed according to the 10–20 international electrodes placement system [42]. The impedance was kept under 10 kΩ. The examinations were performed with a reference A2 electrode placed on the right ear. The sampling frequency was 500 Hz. All signals were acquired using the ExG-32 headbox manufactured by ELMIKO BIOSIGNALS (Milanówek, Poland). The local Bioethical Commission of the Copernicus University in Toruń approved the experiments. Participants had normal or corrected-to-normal vision, and were right-handed.

The study was performed using the resting-state paradigm with eyes open as the EEG signal is heritable [43,44], which suggests that the resting-state EEG consists of persistent individual features that can be used in biometric recognition. This paradigm has always accompanied the development of neuroscience. Since the seminal discovery of fMRI resting-state connectivity [45], the resting-state in fMRI and EEG/MEG studies is at the heart of human connectome analyses as it reflects the relatively natural state of mind. Compared to other paradigms, it is practical in real-world applications. During acquisition, it does not require additional software, and the participant does not need to perform intentional actions, which can be exhausting if carried out regularly.

### 2.2. Preprocessing and Features Extraction

For each examination, we analyzed 3 min of data after the first 30 s. The data were filtered in the frequency range of 0.2–70 Hz, and the Notch filter (50 Hz) was applied. All analyses were performed in MATLAB 2020b. 

To improve the signal-to-noise ratio (SNR), we re-referenced channels to the Common Average Reference (CAR) composed of the 19 electrodes [46]:

(1) $$x_{i,ch}=x_{i,ch}-\frac{1}{CH}\sum_{ch=1}^{CH}\left(x_{i}\right),$$

where *x* is the analyzed data vector, *i* is the sample number, and *ch* is the channel number.

Continuing our previous studies [41], every examination was divided into 7.5 s segments. Welch’s power spectral density was estimated for each frequency in the 1–45 Hz range. We used a 1 s sliding Hamming window with 0.5 s overlap. The analyses were performed on both the raw PSD estimate coefficients and converted to the decibel scale:

(2) $$P_{dB\;i,ch}=10\times log_{10}P_{i,ch},$$

where *P* is the PSD estimate coefficients, *i* is the frequency bin, and *ch* is the channel number.

**Figure 1:**
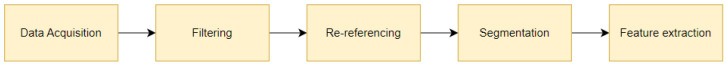
Signal preprocessing path with feature extraction.

The described preprocessing procedure is presented in Figure 1.

### 2.3. Data Set Creation and Results Evaluation

Following the results of our previous studies [41], we decided to use a feed-forward artificial neural network [47] with one hidden layer and two output neurons. In the output layer, we used the linear transfer function, and in the hidden layer, we used a sigmoidal hyperbolic activation function. Our previous study [41] confirmed that one hidden neuron in the hidden layer should be sufficient for verification. This was also confirmed in the current study. We performed a two-class classification [18]. A dedicated neural network trained with the Levenberg–Marquardt backpropagation algorithm [48] was created for each participant.

Since this is an analysis of the verification for each person, separate training and testing data sets were created. The core part of analyses always included the last five recordings. To stratify the data set, an equal number of impostor segments were randomly drawn from the impostor data set.

**Figure 2:**
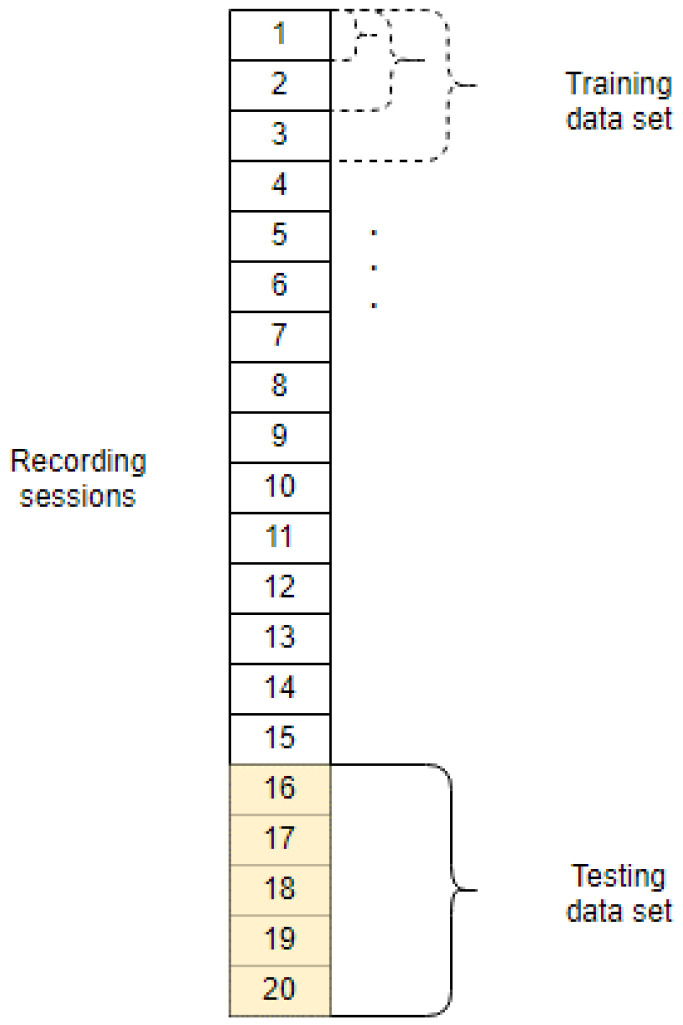
Data-division diagram for the core part of analyses for one participant. Dashed lines for the training data set stand for a different number of recording sessions used to train the model.

By increasing the number of recordings used for training from one to 15, we analyzed their impact on the test results. The data-division diagram is presented in Figure 2. In the training data set, we performed 8-fold cross-validation for each number of recordings. Each time, 10 neural networks were created, from which the best one was selected based on the result of the validation data. Since the genuine and impostor sets are unequal, we performed stratified cross-validation to make the results non-biased. The final test results were averaged. 

The results of the EEG-based verification were assessed using the following classification measures: accuracy (*ACC*), sensitivity (*SEN*), specificity (*SPEC*), and precision (*PREC*):

(3) $$ACC=\frac{TP+TN}{TP+FN+FP+TN},$$

(4) $$SEN=\frac{TP}{TP+FN},$$

(5) $$SPEC=\frac{TN}{FP+TN},$$

(6) $$PREC=\frac{TP}{TP+FP},$$

where *TP* (True Positive) is the number of segments correctly recognized as a claimant (the person who the neural network had learned to recognize); *TN* (True Negative) is the number of segments correctly rejected as an impostor (an unauthorized person); *FP* (False Positive) is the number of segments that improperly recognized an impostor as a claimant; and *FN* (False Negative) is the number of segments incorrectly rejected as a claimant. In this paper, sensitivity is considered as the ability of the algorithm to correctly verify the person as the claimant; specificity as the ability to properly recognize the impostor; accuracy as the overall factor of correctly recognized segments; and precision as the factor of correctly recognized claimant segments from all segments recognized as the claimant.

## 3. Results of the Experiment

The studies on EEG-based human recognition often use databases containing a limited number of distinct, separated-in-time sessions. However, models based on one recording session cannot provide proper model evaluation. The EEG signals are vulnerable to interferences, and while conducting training and testing based on only one recording session, there is a possibility that the signals can be heavily noised or distorted by environmental or technical interferences. Furthermore, in extreme cases, the learning mechanism may adapt to these interferences instead of EEG patterns. Moreover, it can adapt to the spontaneous brain activity. Thus, among other aspects, we decided to analyze the influence of the number of recording sessions used for training on the performance of EEG-based recognition. In our experiments, all sessions for each participant are delivered from 20 distinct sessions separated in time. The crucial aspect of this evaluation is that the test data sets are completely separated in time from the training ones. This approach should exclude the impact of additional unpredictable variables such as temporal daily EEG changes or technical aspects and interferences. In real-life applications of such a system, it is crucial to properly verify the distinct signals based on the smallest possible number of training sessions. We verified our hypothesis by analyzing the impact of an increasing number of sessions used for training on the accuracy results of the last five sessions of each participant.

### 3.1. Training Sessions: 1–15, Testing Sessions: 16–20

In the first series of experiments, we studied the impact of the number of sessions in the training data set on the classification measures which reflect the performance of the model. To determine the number of sessions after which there is no significant improvement, we were changing the number of recording sessions in the training data set from one to 15. Meanwhile, we evaluated how different methods of normalization of PSD estimate coefficients would affect the results. Although we analyzed many methods of normalizing PSD, the best results were obtained for converting it to the decibel scale. That comparison is also one of the original results that we have not seen before in EEG-based recognition. The statistical classification measures (3)–(6) were tested each time on the last five recordings. In the first approach (I), we analyzed the raw PSD estimate coefficients. In the second approach (II), the PSD estimate coefficients were converted to the decibel scale.

**Figure 3:**
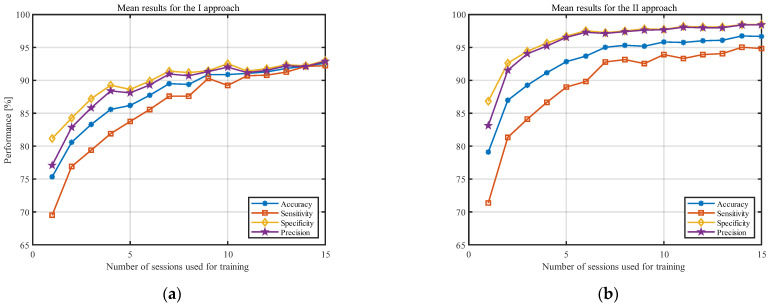
Results for classification measures, according to the number of sessions used for training, averaged over all individuals for the (**a**) I approach and (**b**) II approach.

**Figure 4:**
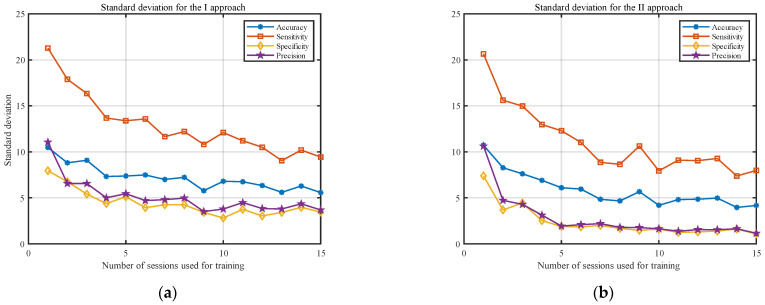
Results for standard deviation, according to the number of sessions used for training, averaged over all participants for the (**a**) I approach and (**b**) II approach.

Figure 3 shows the averaged results (over all participants) for both approaches. The accuracy of the model is presented with the blue line, sensitivity with the red line, specificity with the yellow line, and precision with the purple line. It should be noted that, to highlight the differences in both approaches, the y-axis was limited to 65–100%. The standard deviations of the analyzed statistical measures are presented in Figure 4. The y-axis in this figure was limited to 0–25%.

The results presented in Figure 3 and Figure 4 indicate that, as the number of training sessions increases, the classification measures tend to improve for both approaches. The performance measures increase, and the standard deviations decrease significantly. This is particularly noticeable for the small number of sessions (1–8). One of the most important conclusions is that PSD estimate coefficients provide better results for all considered classification measures when converted to the decibel scale. Moreover, the results for the II approach tend to stabilize faster and show less fluctuation between the obtained results in the last sessions. The results from Figure 3 and Figure 4 are also presented in Table 1 and Table 2 to enable a more detailed comparison. 

For the clarity of the paper, we decided to only consider approach II going forward. This choice is justified because the classification measures obtained for the PSD estimate coefficients converted to the decibel scale provided significantly better results (for the accuracy, the *p*-value obtained with the Wilcoxon Rank Sum Test is equal to 0.00015).

To reject the hypothesis that the observed improvement of classification measures with the increasing number of sessions is the result of only an increase in the size of the data training sets, we analyzed how data variety influences a single training session. For each person, we created a training data set with a size corresponding to a single training session (24 segments). We added one to two segments out of 15 training examinations and analyzed the results for five test sessions. An equal number of impostor segments were randomly drawn from the impostor data sets for the training and testing sets. The obtained accuracy was 88.2 ± 6.8%; sensitivity was 90.1 ± 10.1; specificity was 86.3 ± 6.4%; and precision was 87.4 ± 5.7%. 

### 3.2. Optimal Number of Training Sessions

**Figure 5:**
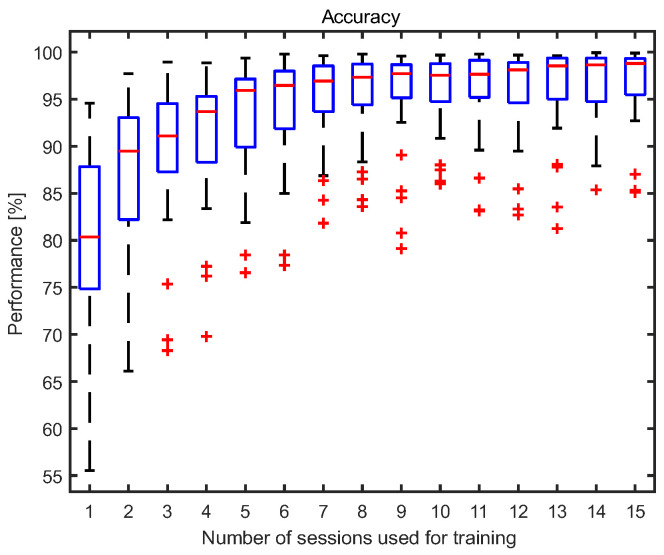
The box plots of accuracy versus the number of sessions used for training.

Based on the results presented in Section 3.1, after a certain number of training sessions, there is no significant improvement in the values of classification measures. The box plots of accuracy (2) obtained for participants versus the number of sessions used for training are visualized in Figure 5. In each box, the median is marked with a red line and the 25th and 75th percentiles with bottom and upper box edges. The outliers are plotted with a cross symbol, and the whiskers are extended to the most extreme values, not considered as outliers.

To compare the results for a different number of training sessions, we first verified whether the obtained data came from the standard normal distribution by performing the one-sample Kolmogorov–Smirnov test. The *p*-values obtained are presented in Table 3. The test sample was the vector of averaged accuracy obtained for each participant.

Next, we analyzed how the number of sessions used in the training data set affected the obtained results. Since we do not have sufficient evidence to reject the hypothesis that the data from all observations follow the normal distribution, we performed the Wilcoxon Rank Sum Test to compare the accuracy values for the different numbers of sessions used to create the training data set. The results are presented in Table 4. The multiple comparisons of accuracy showed no evidence of a significant difference between eight or more sessions used for training, which suggests that there is no need to further increase the number of sessions in the training data set to more than eight.

### 3.3. Results for Training Sessions 1–8 and Testing Sessions 9–20

**Figure 6:**
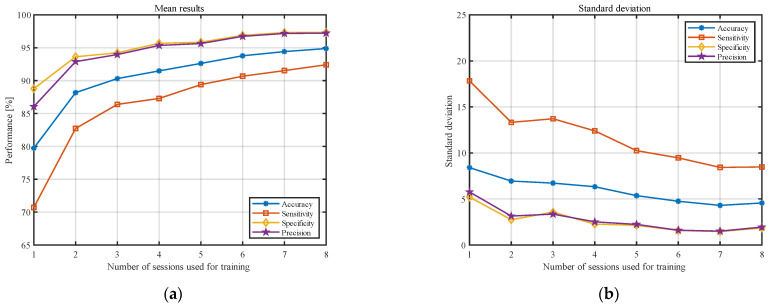
Results for 12 test sessions and the training ones varying from one to eight. (**a**) Classification measures. (**b**) Standard deviations averaged over all individuals.

Since we cannot prove differences between using eight sessions or more, we decided to study the behavior of this number of sessions in the case of an increased number of examinations in the testing data sets. Therefore, we changed the number of examinations included in the training data set from one to eight and used the data from the last 12 recording sessions as the testing data set. The obtained averaged results of the classification measures and standard deviations are presented in Figure 6. The corresponding results are also presented in Table 5 in numerical form.

To extend our analysis, we investigated whether an even greater increase in the testing data set would affect the analyzed classification measures. For this reason, we also evaluated the model’s performance using one training session and 19 testing sessions. The achieved accuracy was 81.1 ± 8.3%; sensitivity was 74.7 ± 15.5%; specificity was 87.4 ± 5.6%; and precision was 85.8 ± 6.8%. There was no evidence for a significant difference between the accuracy obtained for one training session and 19 testing sessions and the accuracy obtained for one training session and five testing sessions (the *p*-value obtained with the Wilcoxon Rank Sum Test is equal to 0.62; see Table 2 for details). 

### 3.4. Simulated Impostor Attack

In the final part of our analysis, we tested the robustness of the proposed EEG-based verification approach by simulating an attack by external impostors. For this, we used 23 recordings of additional subjects who were not involved in training the networks in any way. Our goal was to confirm that these external subjects would be recognized as impostors and, thus, would not introduce any additional external impact on the results. 

**Figure 7:**
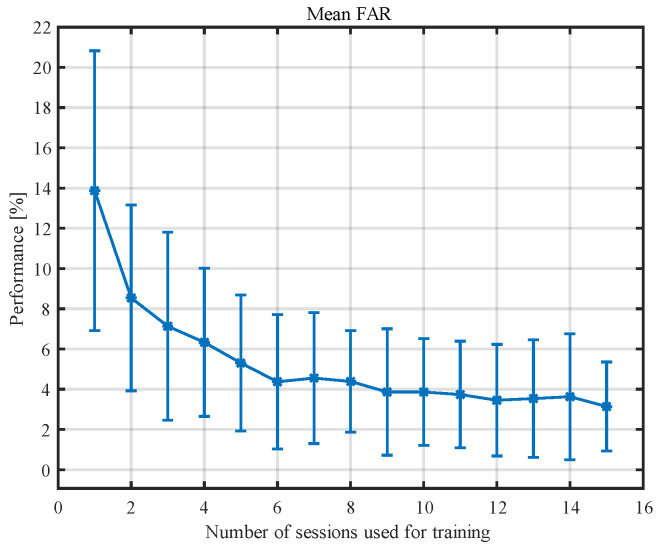
The percentage of successful impostor attacks for different numbers of training recordings.

We conducted this analysis using five examinations for testing and varying the number of training sessions from one to 15. In total, we extracted 552 attack attempts (23 impostors, 24 attempts from 7.5 s segments) from the additional 23 recordings, which were introduced to the dedicated neural network for each participant (29 in total). This resulted in 128,064 attack attempts (552 attack attempts for each of 29 participants with eight networks resulting from cross-validation). The results for the different numbers of sessions used for training are presented in Table 6 and in Figure 7.

Once again, we used the Wilcoxon Rank Sum Test to compare the average number of non-rejected impostors between different numbers of training sessions. The results are presented in Table 7. The multiple comparisons of accuracy showed no evidence for a difference between six or more sessions used for training. The only exception was between eight sessions and 15 sessions.

## 4. Discussion

This paper focused on analyzing the impact of the number of EEG distinct recording sessions used for training on the results of spectral-based method for EEG-based people verification of the claimed identity. For 29 adult participants, we recorded 20 examinations, each performed on a different day. In our studies, we also used 23 single recordings from an additional 23 subjects. In total, for our research, we used 603 examinations, which outperformed other similar studies in terms of the quantity of the database. Initially, we analyzed the results for the two approaches: in the first, we evaluated the features based on the raw PSD estimate coefficients, and in the second, the PSD estimate coefficients were converted to the decibel scale. The assessment was based on the standard classification measures—accuracy, specificity, sensitivity, and precision—as described in Section 2.3. The results showed that converting the PSD estimate coefficients to the decibel scale improved the results for all measures regardless of the number of examinations used for training. The average results increased and their standard deviation decreased; for example, for 15 training sessions, accuracy increased from 92.6 ± 5.6% to 96.7 ± 4.2%, sensitivity increased from 92.2 ± 9.4% to 94.8 ± 8.0%, specificity increased from 93.0 ±3.5% to 98.5 ± 1.1%, and precision increased from 92.9 ± 3.7% to 98.5 ± 1.1% (see Table 1 and Table 2 for comparison).

Observing the results obtained for the classification measures in Figure 3 and Figure 4, one can see that they improve with the number of examinations used for training. However, after a certain number of recordings used for training, this improvement became statistically insignificant. Our analysis showed that, for five examinations used for testing, there is no statistical difference between eight and 15 recordings used for training. For eight training and five testing examinations, the following results were obtained: accuracy: 95.3 ± 4.7%; sensitivity: 93.2 ± 8.6%; specificity: 97.5 ± 1.7%; and precision: 97.4 ± 1.8%. For comparison, the results for 15 training and five testing recordings were as follows: accuracy: 96.7 ± 4.2%; sensitivity: 94.8 ± 8.0%; specificity: 98.5 ± 1.1%; and precision: 98.5 ± 1.1%. Although the results for more sessions seem to be better in all cases, these differences were not statistically significant. 

To our best knowledge, this kind of analysis has not been attempted previously. Although the presented results are for the resting-state, they may serve as a baseline for other paradigms. The problem of using data sets with an insufficient number of distinct recordings is still a concern [17,19,31,32,39,40,49,50,51]. Using data from the same recording session to train and test the model may result in changes in the EEG and its spectral characteristics not being captured. We clearly showed that the EEG changes over time. If more than one recording session is used for training, the results are better because the model learns the nonstationary behavior of the characteristics.

Particular attention should be paid to sensitivity which, consistently, in each case, achieved the worst results among all classification performance measures. At the same time, after a relatively small number of sessions, specificity and precision reached saturation levels, after which there was no significant improvement. In addition, concerning the other measures, a higher standard deviation was achieved in sensitivity. This may suggest some intra-individual changes between sessions which are so large that the system more often falsely rejects the segments of genuine claimants. This result is not surprising, since EEG tends to change slightly on a daily basis, overlap with muscle activity [52], and may be affected by the emotional state [3]. From the biometrics perspective, this behavior seems appropriate: when one is trying to gain access to a secure place, it is better for the system to reject a genuine claimant than to accept an unauthorized person.

Nevertheless, this suggests that more studies are needed to properly evaluate the performance of biometric systems based on EEG. Otherwise, there is a danger that the system will not correctly recognize people and reject impostors. Nevertheless, the achieved results are promising despite using simple features based on the PSD estimate coefficients.

We also verified how the observed improvement of the model performance with increasing numbers of sessions was due to an increase in the number of segments in the training data set or an increase in their diversity (see Section 3.1. for details). The obtained outcomes (ACC: 88.2 ± 6.8%, SEN: 90.1 ± 10.1%, SPEC: 86.3 ± 6.4%, PREC: 87.4 ± 5.7%) were lower than those obtained for all segments from 15 training sessions (ACC: 96.7 ± 4.2%, SEN: 94.8 ± 8.0%, SPEC: 98.5 ± 1.1%, PREC: 98.5 ± 1.1%). However, they were higher than those using data from only one training session (ACC: 79.1 ± 10.7%, SEN: 71.4 ± 20.6%, SPEC: 86.8 ± 7.4%, PREC: 83.1 ± 10.6%). The difference is particularly noticeable in sensitivity, where the obtained sensitivity was 71 ± 20.6% using only one training session, without information from the others.

Since eight recording sessions for the training seemed sufficient to carry out the verification, we studied how the outcomes would be affected by increasing the test set. For this reason, the last 12 recordings were included in the testing data set. Then, the following results were obtained: accuracy: 94.9 ± 4.6%; sensitivity: 92.4 ± 8.5%; specificity: 97.3 ± 1.8%; and precision: 97.2 ± 1.9%. These outcomes are almost identical to those obtained using eight recordings and a smaller testing set (see Table 2 for details). In addition, following this logic, we also decided to study the behavior of the system with only one training examination and 19 test recordings for the analysis. For the obtained results (accuracy: 81.1 ± 8.3%; sensitivity: 74.7 ± 15.5%; specificity: 87.4 ± 5.6%; precision: 85.8 ± 6.8%), we did not find evidence for the significant difference. The obtained results suggest that five studies may be sufficient for testing.

The results obtained in the simulated attack by impostors correspond to the specificity obtained for five testing examinations and the number of training sessions varying from one to 15. As the number of sessions increases, the value of specificity decreases. The obtained number for the specificity was 98.5 ± 1.1%, while the percentage of successful impostor attacks over all attack attempts was 3.1 ± 2.2%. The multiple comparisons of accuracy showed that six examinations were not significantly different from 15 examinations used for training (see Table 7 for details). This number is lower than that calculated based on classification measures, but this should not be surprising. FAR is associated with specificity which, in our studies, stabilized quite quickly, while the deficiencies in stability primarily concerned sensitivity. However, the FAR coefficient still needs to be reduced. This may be achieved by combining this method with another one; e.g., entropy or connectivity measures [23,40]. In this paper, we assessed the results of people verification obtained for analysis of 7.5 s EEG segments, averaged for all participants. Increasing the length of the analyzed segments as well as the number of segments used for verification should decrease the FAR coefficient. The results may also benefit from analyzing the impact of the EEG sub-bands and limiting the method only to the sub-bands that have the greatest impact on the verification results, which would allow for further noise reduction. It should be noted that the results achieved for EEG-based biometrics are still slightly worse compared to the other biometrics methods developed over many decades, but the research on EEG biometrics is still in its fledgling state [53].

## 5. Conclusions

One of the challenges in biometrics using methods based on the EEG signal analysis is to train mechanisms for automatic people verification in the shortest possible time while ensuring sufficient verification results. In most of the results published in the literature, with only a few exceptions, training and testing data sets come from the same EEG recordings, which has several drawbacks:

1. Using the same recordings for training and testing may cause data leakage, resulting in false high classification accuracy.
2. One session may not reflect the technical and environmental conditions encountered in various practical situations as the practical application of the considered EEG-based method involves multiple sessions that bring additional variance due to differences in electrode montage, impedance, and the mental state of a subject. None of these sources of variance is accessible to machine learning procedures worked out from one session’s data set.
3. In practical applications, some electrodes may not contact properly and not always in the same way.

This study investigated how different numbers of distinct recording sessions would affect EEG-based verification. One of the main conclusions is that increasing the number of test sessions did not significantly affect the result obtained. It turned out to be essential to use more training sessions. This result was due to verification sensitivity, which appears to be less stable than specificity. Our analysis showed that the results improved as the number of training sessions increased. However, more than eight distinct recording sessions for the proposed approach did not make a significant difference. In future studies, we plan to develop a more advanced method for EEG-based verification and reduce the number of electrodes. Moreover, due to the EEG variability, it is essential to perform similar studies under heterogenous conditions.

## Figures and Tables

**Table 1:** Classification measures averaged over all participants obtained for the I approach, in which the raw PSD estimate coefficients were analyzed.

Session No.	1	2	3	4	5	6	7	8	9	10	11	12	13	14	15
ACC [% ± SD]	75.3 ±10.5	80.6 ±8.8	83.3 ±9.1	85.6 ±7.3	86.2 ±7.4	87.7 ±7.5	89.5 ±7.0	89.4 ±7.2	90.9 ±5.8	90.9 ±6.8	91.1 ±6.8	91.3 ±6.3	91.8 ±5.6	92.2 ±6.3	92.6 ±5.6
SEN [% ± SD]	69.5 ±21.3	76.9 ±17.9	79.4 ±16.3	81.9 ±13.7	83.8 ±13.4	85.6 ±13.6	87.6 ±11.7	87.6 ±12.2	90.3 ±10.8	89.2 ±12.1	90.7 ±11.2	90.8 ±10.5	91.3 ±9.0	92.1 ±10.2	92.2 ±9.4
SPEC [% ± SD]	81.2 ±7.9	84.3 ±6.8	87.2 ±5.4	89.3 ±4.4	88.6 ±5.1	89.9 ±3.9	91.4 ±4.2	91.2 ±4.2	91.5 ±3.4	92.5 ±2.8	91.4 ±3.7	91.8 ±3.0	92.4 ±3.4	92.2 ±4.0	93.0 ±3.5
PREC [% ± SD]	77.1 ±11.1	82.9 ±6.6	85.8 ±6.6	88.4 ±5.0	88.1 ±5.5	89.3 ±4.7	91.0 ±4.8	90.7 ±5.0	91.3 ±3.5	92.0 ±3.8	91.2 ±4.5	91.5 ±3.8	92.2 ±3.8	92.1 ±4.4	92.9 ±3.7

**Table 2:** Classification measures averaged over all participants obtained for the II approach, in which the PSD estimate coefficients converted to the decibel scale were analyzed.

Session No.	1	2	3	4	5	6	7	8	9	10	11	12	13	14	15
ACC [% ± SD]	79.1 ±10.7	87.0 ±8.3	89.3 ±7.6	91.2 ±6.9	92.8 ±6.1	93.7 ±6.0	95.0 ±4.8	95.3 ±4.7	95.2 ±5.7	95.8 ±4.2	95.8 ±4.8	96.0 ±4.9	96.1 ±5.0	96.8 ±4.0	96.7 ±4.2
SEN [% ± SD]	71.4 ±20.6	81.3 ±15.6	84.1 ±15.0	86.7 ±13.0	89.0 ±12.3	89.8 ±11.0	92.8 ±8.9	93.2 ±8.6	92.6 ±10.6	93.9 ±7.9	93.3 ±9.1	93.9 ±9.0	94.1 ±9.3	95.0 ±7.4	94.8 ±8.0
SPEC [% ± SD]	86.8 ±7.4	92.6 ±3.7	94.4 ±4.4	95.7 ±2.5	96.7 ±1.9	97.6 ±1.8	97.3 ±2.0	97.5 ±1.7	97.8 ±1.5	97.8 ±1.6	98.2 ±1.2	98.1 ±1.3	98.1 ±1.4	98.5 ±1.6	98.5 ±1.1
PREC [% ± SD]	83.1 ±10.6	91.5 ±4.7	94.0 ±4.3	95.2 ±3.1	96.5 ±1.9	97.3 ±2.1	97.1 ±2.2	97.4 ±1.8	97.6 ±1.8	97.7 ±1.6	98.1 ±1.4	98.0 ±1.5	98.0 ±1.5	98.4 ±1.6	98.5 ±1.1

**Table 3:** Results of the Kolmogorov–Smirnov test. The *p*-values lower than 0.05 (which means they do not come from the standard normal distribution) are marked in red.

Session No.	1	2	3	4	5	6	7	8	9	10	11	12	13	14	15
*p*-val.	0.613	0.317	0.276	0.153	0.141	0.197	0.138	0.123	0.027	0.092	0.040	0.012	0.028	0.013	0.028

**Table 4:** Results of the Wilcoxon Rank Sum Test. The *p*-values lower than 0.05 (which means significant differences between groups) are marked in red.

Session No.	1	2	3	4	5	6	7	8	9	10	11	12	13	14	15
1		0.002	<0.001	<0.001	<0.001	<0.001	<0.001	<0.001	<0.001	<0.001	<0.001	<0.001	<0.001	<0.001	<0.001
2			0.343	0.020	0.001	<0.001	<0.001	<0.001	<0.001	<0.001	<0.001	<0.001	<0.001	<0.001	<0.001
3				0.202	0.042	0.006	0.001	<0.001	<0.001	<0.001	<0.001	<0.001	<0.001	<0.001	<0.001
4					0.213	0.056	0.005	0.002	0.001	0.001	<0.001	<0.001	<0.001	<0.001	<0.001
5						0.494	0.109	0.048	0.040	0.019	0.011	0.003	0.003	0.001	0.001
6							0.343	0.231	0.150	0.120	0.078	0.040	0.017	0.005	0.007
7								0.738	0.549	0.460	0.273	0.197	0.077	0.033	0.047
8									0.721	0.597	0.460	0.259	0.234	0.090	0.126
9										0.774	0.715	0.388	0.247	0.162	0.186
10											0.791	0.455	0.384	0.191	0.216
11												0.732	0.499	0.269	0.401
12													0.663	0.465	0.414
13														0.852	0.834
14															1.000
15															

**Table 5:** Classification measures averaged over all participants obtained for the number of training sessions varying from 1 to 8 and the 12 testing sessions.

Session No.	1	2	3	4	5	6	7	8
ACC [% ± SD]	79.7 ±8.4	88.2 ±6.9	90.3 ±6.7	91.5 ±6.3	92.6 ±5.4	93.8 ±4.8	94.4 ±4.3	94.9 ±4.6
SEN [% ± SD]	70.7 ±17.8	82.7 ±13.3	86.4 ±13.7	87.3 ±12.4	89.4 ±10.3	90.7 ±9.5	91.5 ±8.4	92.4 ±8.5
SPEC [% ± SD]	88.8 ±5.2	93.6 ±2.7	94.2 ±3.6	95.7 ±2.3	95.8 ±2.1	96.9 ±1.6	97.3 ±1.5	97.3 ±1.8
PREC [% ± SD]	86.1 ±5.8	92.9 ±3.1	94.0 ±3.4	95.4 ±2.5	95.6 ±2.2	96.7 ±1.6	97.2 ±1.5	97.2 ±1.9

**Table 6:** The percentage of successful impostor attacks over all attack attempts (False Acceptance Rate, FAR).

Session No.	1	2	3	4	5	6	7	8	9	10	11	12	13	14	15
FAR [% ± SD]	13.9 ±7.0	8.5 ±4.6	7.1 ±4.7	6.3 ±3.7	5.3 ±3.4	4.4 ±3.3	4.6 ±3.3	4.4 ±2.5	3.9 ±3.1	3.9 ±2.7	3.7 ±2.7	3.5 ±2.8	3.5 ±2.9	3.6 ±3.1	3.1 ±2.2

**Table 7:** Results of the Wilcoxon Rank Sum Test for the simulation of impostor attack. The *p*-values lower than 0.05 (which means significant differences between groups) are marked in red.

Session No.	1	2	3	4	5	6	7	8	9	10	11	12	13	14	15
1		0.002	<0.001	<0.001	<0.001	<0.001	<0.001	<0.001	<0.001	<0.001	<0.001	<0.001	<0.001	<0.001	<0.001
2			0.111	0.047	0.002	<0.001	<0.001	<0.001	<0.001	<0.001	<0.001	<0.001	<0.001	<0.001	<0.001
3				0.803	0.140	0.008	0.015	0.012	0.003	0.002	0.002	<0.001	<0.001	<0.001	<0.001
4					0.237	0.031	0.042	0.042	0.006	0.006	0.007	0.002	0.001	0.002	0.001
5						0.166	0.331	0.320	0.044	0.050	0.055	0.014	0.021	0.021	0.004
6							0.709	0.441	0.504	0.680	0.555	0.297	0.308	0.283	0.237
7								0.901	0.305	0.423	0.320	0.124	0.179	0.202	0.084
8									0.240	0.308	0.191	0.074	0.111	0.176	0.023
9										0.709	0.938	0.780	0.780	0.658	0.732
10											0.756	0.446	0.534	0.428	0.290
11												0.732	0.774	0.709	0.555
12													0.803	0.876	0.810
13														0.901	0.641
14															0.669
15															

## Data Availability

Data available on request.
